# Health-related quality of life analysis in differentiated thyroid carcinoma patients after thyroidectomy

**DOI:** 10.1038/s41598-020-62731-3

**Published:** 2020-04-01

**Authors:** Jie Li, Bo Zhang, Yang Bai, Yonghong Liu, Buyong Zhang, Jian Jin

**Affiliations:** The Fourth Department of Thyroid and Breast Surgery, Cangzhou Central hospital, Hebei, China

**Keywords:** Cancer, Surgical oncology

## Abstract

Although differentiated thyroid carcinoma (DTC) has a good prognosis and survival rate, long-term medication and recurrence monitoring might be needed. The factors that affect postoperative health-related quality of life (HRQoL) in patients with DTC in different regions remain unclear or conflicting. The purpose of this study was to assess the factors that influence the HRQoL of DTC patients after surgery. This study selected 174 patients with DTC who underwent thyroidectomy. Additionally, 174 participants who were matched by age, gender, and socioeconomic status were recruited from the population as the control group. Both the DTC and control population groups were invited to answer the HRQoL questionnaire SF-36. Scores on seven domains of the HRQoL including role-physical (RP), bodily pain (BP), general health (GH), vitality (VT), social functioning (SF), role-emotional (RE), and mental health (MH), were significantly lower for DTC patients than for the control population. The patients with no comorbidities had much higher scores on the 8 domains of the SF-36 than DTC patients with two or more comorbidities (all P < 0.05). Hypertension, diabetes and depression were the predictive factors of a poor Physical Component Summary (PCS) score and diabetes and depression were predictive factors of the Mental Component Summary (MCS) score at one year of follow-up (all P < 0.05). HRQoL is significantly influenced by many sociodemographic and clinical factors. Hypertension, diabetes and depression had a negative impact on HRQoL in DTC patients. More attention and targeted intervention should be given to DTC patients after surgery to improve quality of life.

## Introduction

Thyroid cancer is the most common malignant tumor of the endocrine system and the head and neck. New cases of thyroid cancer account for approximately 1–5% of all cancers each year^1^. In the past 20 years, the incidence of thyroid cancer has increased each year, causing widespread concern. Differentiated thyroid carcinoma (DTC) frequently occurs in young and middle-aged women^2^. This type of thyroid carcinoma has clinical features of low malignancy, good differentiation, and a slow growth rate^3,4^. However, lymph node metastases can occur months to years after diagnosis, and timely diagnosis and appropriate treatment can result in a favourable outcome.

The recommended clinical treatments of differentiated thyroid cancer are subtotal thyroidectomy, total thyroidectomy, radioactive iodine therapy (RAI) and long-term thyroid hormone replacement therapy^5,6^. These treatments can reduce the probability of cancer recurrence, and more than 85% of patients with differentiated thyroid cancer have a good prognosis according to the 10-year survival rates. Current guidelines recommend radioactive iodine ablation after initial thyroidectomy for high-risk patients. For low-risk DTC patients, the recommended surgical approach is unilateral thyroidectomy or total thyroidectomy. Total resection in low-risk DTC patients may be considered overtreatment. Although the advantage of total thyroidectomy is reduction in the local recurrence rate, the risk of parathyroid injury and laryngeal nerve injury, which greatly impact a patient’s HRQoL, is doubled^7^. Patients require lifelong thyroid hormone replacement therapy, especially after total thyroidectomy. In most cases, suppressing thyroid-stimulating hormone (TSH) using high-dose thyroid hormone replacement therapy can cause side effects, such as osteoporosis, atrial fibrillation, strokes and fracture^8^. Multiple treatments indeed improve the survival time and prognosis of patients with DTC, but their daily lives are also changed in many ways, including their psychological stress, social roles, and interpersonal communication^9^. Currently, the medical model has shifted from a biomedical model to a bio-psycho-social model. Therefore, the evaluation of surgical outcomes has expanded from clinical symptom improvement to the overall improvement in physical and mental health. Many clinical studies have also found that thyroid cancer patients have differences in health-related quality of life (HRQoL) after treatment^10–12^. Therefore, identifying the influencing factors that cause differences in health-related quality of life is important to further improving the treatment effect and patient satisfaction.

Due to the continuous development of technology in the medical field, especially for cancer therapies, improvement of the postoperative health-related quality of life is the key to clinical treatment. Health-related quality of life (HRQoL) is a multidimensional concept that includes symptoms of disease or the health condition, treatment side effects, and functional status across physical, social, and mental health life domains. In recent years, several studies have used health-related quality of life as an evaluation criterion for clinical efficacy^13,14^. However, few studies have evaluated the effects of surgery and radioiodine on health-related quality of life in patients with differentiated thyroid carcinoma^15,16^. The SF-36 is the most extensively validated and used instrument for measuring HRQoL. HRQoL is measured by a questionnaire, and the answers to items are converted into numerical values. Three domains, physical, psychological, and social, are the main factors in the definition of HRQoL. Many cancer clinical trials have also confirmed that pathological staging, treatment programs, treatment-related symptoms, depression and other mental disorders can affect the quality of life of patients^17,18^. Therefore, the purpose of this study was to investigate the differences in postoperative health-related quality of life in patients with differentiated thyroid cancer using the SF-36 quality of life scale. Furthermore, we aimed to explore the influencing factors of health-related quality of life in DTC patients after surgery.

## Methods

### Patients and the control population sample

This is a population-based, a single-centre, cross-sectional cohort study. From June 2015 to July 2016, 174 patients with differentiated thyroid cancer who underwent thyroidectomy and met the eligibility criteria were recruited in the study. All patients underwent a near total thyroidectomy, followed by postoperative radioiodine ablation therapy with I-131 (RAI) and TSH suppressive T4 therapy as initial treatment. The inclusion criteria for this study were (i) patients who were older than 18 years of age with a diagnosis of differentiated thyroid cancer (medium risk and high risk); (ii) subjects who were able to complete the SF-36 questionnaire and a follow-up survey at a minimum of 1 year follow-up survey immediately after surgery. The exclusion criteria included benign thyroid nodules and other types of thyroid carcinoma, patients younger than 18 years of age, patients unable to complete the SF-36 questionnaire or who could not complete the one-year follow-up survey. RAI treatment is selective used in DTC treatment guidelines influencing by multiple factors. RAI treatment may have an impact on the quality of life of DTC patients. Therefore, all patients selected in this study were treated with RAI after surgery. The 174 volunteers in the control group included patients who visited our hospital for routine physical examinations during the same time period. The recruited volunteers were matched by age, gender, ethnicity, marital status, educational level, yearly income, work status, hypertension or diabetes status, and alcohol or smoking consumption, as much as possible. Based on the above parameters, we attempted to identify healthy volunteers with parameters similar to those of DTC patients. The propensity score matching method was then used to screen the appropriate control population. The education level was categorized as low (less than secondary education) or high (higher education and postgraduate education). The subjects in the control group only completed one SF-36 questionnaire. The questionnaire was conducted using traditional paper and pencil self-administration methods. Researchers presented paper questionnaires to patients in person and asked them to complete them by hand and return them to the researchers.

### Health-related quality of life

The Chinese version of the SF-36 questionnaire which has been tested and widely used in China, was used to assess the quality of life of patients in this study^19^. The questionnaire has 8 scale profiles and 36 items, covering the 9 aspects of health related quality of life, including physical functioning (PF), vitality (VT), general health perceptions (GH), role-physical (RP), social functioning (SF), role-emotional (RE), bodily pain (BP) and mental health (MH). Physical and Mental Health Composite Scores (PCS and MCS, respectively) can be calculated by the scores of each question ranging from 0–100. A score of 0 represents the worst, and a score of 100 indicates the best quality of life.

### Socio-demographic variables and comorbidities

When the patients were recruited in the study, the patient’s age, gender, ethnicity, marital status, education level, yearly income, occupation, smoking or alcohol consumption, and menstrual status were collected using a questionnaire. Information about comorbidities that had been diagnosed by a physician, such as atrial fibrillation, hypertension, myocardial infarction, diabetes, chronic obstructive pulmonary disease, stroke, renal failure, osteoporosis, depression, and rheumatoid arthritis, was also collected as much as possible. The patient’s comorbidities were counted. The response alternatives were “Yes” or “No”.

### Ethics

The study protocol was reviewed and approved by the Ethical Committee of the Cangzhou Central Hospital. All experiments were performed in accordance with relevant guidelines and regulations. Written informed consent was obtained from all patients.

### Statistical analysis

Statistical analysis was performed using the SPSS 20.0 statistical software package. Clinical characteristics of patients are described by descriptive statistics. DTC patient scores derived from the SF-36 were compared with mean scores in the multiple parameter-matched control group. The categorical variables were compared between groups using Chi-square tests. Differences in the means of the two groups were analysed with independent samples t-tests. Continuous variables were compared by one-way analysis of variance. The relationships between factors and quality of life were first examined using simple logistic regression. A multiple logistic regression model was used to detect the independent factors associated with quality of life. Differences were considered statistically significant at P < 0.05.

## Results

### Clinical characteristics of the DTC patients and control group

As shown in Table 1, 174 patients and 174 matched control populations took part in the study, and the majority of the DTC patients (75.9%) were women. The average age was 43.3 years, ranging from 20 ~ 74 years. Regarding the education level, 54.6% of the DTC patients had less than 12 years of education, while the remaining DTC patients had college education experience. Individuals with an annual income below 150 thousand RMB accounted for 70.1% of DTC patients. The proportions of DTC patients with hypertension and diabetes were 8.0% and 5.2%, respectively. However, no significant differences were observed between the DTC patients and control population for clinical characteristics. Therefore, the baseline data of the control population was well matched with that of the DTC patients, indicating effective selection of the control population.

**Table 1:** Characteristics of the DTC patients and control group.

		DTC patients	Control population	P
		(n = 174)	(n = 174)	
Age		43.3 ± 12.2	42.8 ± 12.3	0.692
Gender	Male	42	44	0.804
	Female	132	130	
Ethnicity	Han	166	164	0.371
	Others	8	12	
Marital status	Married	159	157	0.711
	Unmarried	15	17	
Educational level	Low	95	91	0.667
	High	79	83	
Yearly income	Low	122	128	0.475
	High	52	46	
Alcohol consumption	Yes	21	22	0.871
	No	153	152	
Smoking	Yes	23	24	0.875
	No	151	150	
Hypertension	Yes	14	13	0.841
	No	160	161	
Diabetes	Yes	9	10	0.813
	No	165	164	
Myocardial infarction	Yes	4	3	0.703
	No	170	171	
Stroke	Yes	4	5	0.736
	No	170	169	
Asthma	Yes	12	11	0.829
	No	162	163	
Kidney disease	Yes	10	12	0.660
	No	164	162	
Depression	Yes	13	10	0.517
	No	161	164	

low income = less than 150 thousand RMB; high income = more than 150 thousand RMB.

### HRQoL at one year of follow-up

The HRQoL of DTC patients was evaluated at one year of follow-up. As described in Table 2, DTC patients showed much lower scores on 7 domains (all domains except for physical functioning (PF)) of the SF-36 (all P < 0.05). No statistically significant difference was found between DTC patients and the control population in the PF domain (P = 0.694). In addition, the PCS and MSC scores of the DTC patients were also lower than those in the control population (all P < 0.05, Table 2).

**Table 2:** SF-36 scores of the DTC patients and control population at one year of follow-up.

	DTC patients	Control population	P
	(n = 174)	(n = 174)	
Physical functioning (PF)	86.0 ± 8.4	86.3 ± 7.9	0.694
Role-physical (RP)	79.5 ± 31.0	86.6 ± 23.8	0.016
Bodily pain (BP)	79.7 ± 23.1	87.1 ± 17.7	<0.001
General health (GH)	61.4 ± 25.3	73.1 ± 22.1	<0.001
Vitality (VT)	62.1 ± 26.7	70.8 ± 22.3	<0.001
Social functioning (SF)	77.2 ± 17.3	84.8 ± 13.2	<0.001
Role-emotional (RE)	74.3 ± 23.6	84.3 ± 22.2	<0.001
Mental health (MH)	71.5 ± 21.8	81.7 ± 20.3	<0.001
PCS	76.8 ± 10.5	83.3 ± 9.9	<0.001
MCS	71.3 ± 10.8	80.4 ± 10.1	<0.001

### The effect of comorbidities on HRQoL at one year of follow-up

Comorbidities had an impact on the HRQoL of DTC patients. Therefore, we analysed the influence of comorbidities on the HRQoL of DTC patients at one year of follow-up. Patients with no comorbidities had much higher scores on the 8 domains of the SF-36 than DTC patients with two or more comorbidities (all P < 0.05, Table 3). With the exception of the social functioning (SF) domain, the scores of the remaining 7 domains were much lower in DTC patients with one comorbidity than in patients with no comorbidity (all P < 0.05). Further comparison between sub-groups also showed that patients with two or more comorbidities had lower SF-36 scores, except for the role-emotional (RE) domain. Furthermore, significant differences were also found between groupsfor the PCS and MCS scores.

**Table 3:** Influence of comorbidities on HRQoL at one year of follow-up.

	Number of self-reported comorbidities			P
	None(90)	One(52)	At least two(32)	
Physical functioning (PF)	91.3 ± 5.9	83.2 ± 4.7*	75.6 ± 6.9*#	<0.001
Role-physical (RP)	94.7 ± 13.2	79.3 ± 20.2*	37.5 ± 12.7*#	<0.001
Bodily pain (BP)	92.1 ± 9.7	79.2 ± 19.9*	49.1 ± 9.6*#	<0.001
General health (GH)	78.0 ± 20.1	49.5 ± 15.6*	33.9 ± 14.2*#	<0.001
Vitality (VT)	78.7 ± 20.5	49.6 ± 19.2*	35.9 ± 19.9*#	<0.001
Social functioning (SF)	83.2 ± 15.7	78.4 ± 10.5	58.2 ± 16.7*#	<0.001
Role-emotional (RE)	83.7 ± 18.9	66.6 ± 24.7*	60.4 ± 23.1*	<0.001
Mental health (MH)	88.3 ± 10.4	62.5 ± 10.3*	39.1 ± 12.1*#	<0.001
PCS	89.0 ± 6.9	72.8 ± 10.9*	49.0 ± 8.5*#	<0.001
MCS	83.5 ± 9.5	64.3 ± 8.2*	48.4 ± 16.6*#	<0.001

All values are given as the mean ± SD. *P < 0.05 vs the no comorbiditiesgroup. # P < 0.05 vs the one comorbidity group.

### Univariate and multivariate analysis of PCS and MCS scores

Univariate and multivariate linear regression were used to estimate the relationships of sociodemographic factors and comorbidities with PCS and MCS scores. According to the mean PCS and MCS scores of DTC patients, the cut-off values of the PCS and MCS scores were 76.8 and 71.3, respectively. Among these factors, hypertension, diabetes, asthma and depression were found to be statistically significant in the univariate analysis of PCS and MCS scores. In the subsequent multivariate analyses, hypertension, diabetes and depression were predictive factors of a poor PCS score, and diabetes and depression were predictive factors of the MCS score at one year of follow-up, as summarized in Table 4.

**Table 4:** Univariate and multivariate analysis of PCS and MCS scores.

	Univariate analysis	Multivariate analysis	P
	P	HR(95% CI)	
**PCS**			
Age <45≥ 45 years	0.578		
Gender (male/female)	0.253		
Ethnicity (Han/other)	0.712		
Marital status (married or unmarried)	0.872		
Educational level (low/high)	0.553		
Yearly income (low/high)	0.441		
Alcohol consumption (yes/no)	0.252		
Smoking (yes/no)	0.859		
Hypertension (yes/no)	0.000	2.939 (1.656–5.217)	0.012
Diabetes (yes/no)	0.002	2.738 (1.243–6.032)	0.008
Myocardial infarction (yes/no)	0.660		
Stroke (yes/no)	0.825		
Asthma (yes/no)	0.038		
Kidney disease (yes/no)	0.137		
Depression (yes/no)	0.033	2.223 (1.231–4.014)	0.001
**MCS**			
Age<45≥ 45 years	0.083		
Gender (male/female)	0.477		
Ethnicity (Han/others)	0.771		
Marital status (married or unmarried)	0.862		
Educational level (low/high)	0.445		
Yearly incoming (low/high)	0.352		
Alcohol consumption (yes/no)	0.646		
Smoking (yes/no)	0.164		
Hypertension (yes/no)	0.002		
Diabetes (yes/no)	0.016	3.012 (1.628–5.223)	0.000
Myocardial infarction (yes/no)	0.642		
Stroke (yes/no)	0.602		
Asthma (yes/no)	0.032	1.906 (1.172–3.228)	0.009
Kidney disease (yes/no)	0.141		
Depression (yes/no)	0.006	2.739 (1.521–4.931)	0.001

## Discussion

Thyroidectomy is still the most commonly performed surgery for any type of thyroid carcinoma^20^. Surgical resection, radioactive iodine therapy, and long-term L-thyroxine replacement therapy can cause various problems in the daily life and psychological state of DTC patients^9^. Although DTC patients have low mortality rates, patients must undergo substantial lifelong surveillance for DTC recurrence^21^. This may produce psychological stress in the patients^22^. The above factors are closely related to the quality of life of postoperative DTC patients. Previous studies have also confirmed that DTC patients reported psychological and physiological problems after surgery^23^. Our study evaluated the relationship between HRQoL and related clinical parameters in a group of patients with papillary thyroid carcinoma after one year of follow-up. First, we compared the difference in HRQoL between 174 DTC patients and 174 control population. The postoperative HRQoL scores of DTC patients were significantly lower than those of the control population in seven domains of the SF-36 scale. Second, we found that DTC patients with one or more co-morbidities had a significantly lower HRQoL score than those without comorbidities. In addition, we identified hypertension and diabetes as risk factors for postoperative HRQoL scores in DTC patients by multivariate regression analysis. Finally, our study is the first to use the SF-36 to assess the HRQoL scores of DTC patients with Han nationality in northern China.

The goal of cancer treatment is not only to prolong survival, but also to maintain and improve the quality of life. Since the 1980s, research on the quality of life of cancer patients has attracted great international attention. Quality of life is the perception of individuals in the context of cultures and value systems in which they live and the state of quality of life is associated with their goals, expectations, standards, and concerns^24^. HRQoL is a multidimensional concept that includes physiological functions, psychological functions, role activities, social adaptability and overall feelings about health^25^. The SF-36 is a universal scale with eight domains for life quality assessment used in tumor patients and the control population. The SF-36 has also widely used in studies of thyroid carcinoma^10^. A previous study reported that external beam radiotherapy impacted the quality of life of patients with advanced thyroid carcinoma^26^. Another study showed that the effect on quality of life was primarily related to the emotional and social impacts of treatment in PTC patients^27^. However, the few previous studies have compared the HRQoL between DTC patients and control participants and few studies have focused on changes in HRQoL after surgery. The results of this study showed that the HRQoL scores were significantly lower in DTC patients than in the control population in 7 domains, namely, RP, BP, GH, VT, SF, RE, and MH. The clinical parameters of the control population were well matched with those of the DTC patients. The reason for the lower scores in multiple domains in DTC patients may be due to the patient’s negative feelings related to their appearance, stigma, physical exhaustion, fear of tumor recurrence, and psychological stress associated with anticancer therapies. In a study by Hedman *et al*., PTC patients showed poorer HRQoL than the control population at 14–17 years after diagnosis and surgery^11^. Another study also showed that disease-free survivors of differentiated thyroid carcinoma (DTC) had a decreased HRQoL in all five functional domains (physical, role, cognitive, emotional, and social) on the EORTC QLQ-C30 compared to the control population based on a survey of 1,000 people^28^.

Comorbidities in DTC patients are also a factor that influence the quality of life. Previous studies have rarely analysed the impact of comorbidities on the postoperative quality of life of DTC patients. Our results revealed that greater number of comorbidities leads to worse HRQoL in DTC patients. For DTC patients with or without comorbidities, the therapeutic regime is still the same the guidelines recommend surgery, I-131 treatment and TSH suppressive T4 therapy. This can ensure that the therapeutic regime does not affect the quality of life in different populations. These comorbidities may be associated with long-term use of L-thyroxine treatment, leading to a decline in the quality of life. Many chronic disease conditions also have a significant negative effect on HRQoL^29^. A study of thyroid cancer, colorectal cancer, and (non-)Hodgkin’s lymphoma patients showed that comorbidities explained more variance in physical and emotional function, pain, fatigue and HRQoL than sociodemographic and cancer characteristics in cancer survivors^30^. Comorbidities had a negative impact on the HRQoL of patients with breast cancer, which increased with time after diagnosis and was strongly associated with cardiovascular disease and depression^31^.

The study also analysed factors that affect the PCS and MCS scores of the SF-36 scale in DTC patients by multivariate regression analysis. Our results showed that hypertension, diabetes and depression were predictive factors of a poor PCS score, and diabetes and depression were predictive factors of the MCS score at one year of follow-up. Hypertension and diabetes are often associated with an increased body mass index, which is often associated with sleep disturbances and anxiety. Blood sugar and blood pressure control and medication compliance were the causes of psychological anxiety in patients. A cancer diagnosis can be considered as a threat of death by patients. Psychological stress and anxiety may be more serious in DTC patients with depression. They are worried about recurrence and death. In addition, depression is also a major contributor to poor quality of life in patients with thyroid carcinoma and other cancers or chronic diseases^32,33^. Therefore, counseling for postoperative psychological problems in DTC patients is the key to improving their quality of life^34^. Additionally, improved the sleep, active participation in social activities, and return to normal social roles can greatly improve the quality of life of DTC patients after surgery.

We acknowledge that our present study has some limitations. The cohort of patients participating in the study was small, and our study was a single-center analysis mainly involving the Han ethnic population of northern China, which may not entirely reflect the Chinese population. The SF-36 scale might not be sufficiently sensitive to capture the perspectives of all DTC patients.

In conclusion, we should consider DTC as a chronic disease in view of the long-term survival of DTC patients. Additional attention and targeted intervention are critical to improving the quality of life of DTC patients after surgery.

## Data Availability

The datasets supporting the conclusions of this article are included within this article and its additional images. Raw data are available from the corresponding author on reasonable request.
